# Cancer cell adaptation to chemotherapy

**DOI:** 10.1186/1471-2407-5-78

**Published:** 2005-07-18

**Authors:** Federica Di Nicolantonio, Stuart J Mercer, Louise A Knight, Francis G Gabriel, Pauline A Whitehouse, Sanjay Sharma, Augusta Fernando, Sharon Glaysher, Silvana Di Palma, Penny Johnson, Shaw S Somers, Simon Toh, Bernie Higgins, Alan Lamont, Tim Gulliford, Jeremy Hurren, Constantinos Yiangou, Ian A Cree

**Affiliations:** 1 Translational Oncology Research Centre, Department of Histopathology, Queen Alexandra Hospital, Portsmouth PO6 3LY, UK; 2 Department of Surgery, Queen Alexandra Hospital, Portsmouth PO6 3LY, UK; 3 Department of Mathematics and Statistics, University of Portsmouth, Buckingham Building, Lion Terrace, Portsmouth PO1 3HE, UK; 4 Department of Radiotherapy and Oncology, Southend Hospital, Prittlewell Chase, Westcliff-on-Sea, Essex SS0 0RY, UK; 5 Department of Radiotherapy and Oncology, St Mary's Hospital, Milton Road, Portsmouth PO3 6AD, UK

## Abstract

**Background:**

Tumor resistance to chemotherapy may be present at the beginning of treatment, develop during treatment, or become apparent on re-treatment of the patient. The mechanisms involved are usually inferred from experiments with cell lines, as studies in tumor-derived cells are difficult. Studies of human tumors show that cells adapt to chemotherapy, but it has been largely assumed that clonal selection leads to the resistance of recurrent tumors.

**Methods:**

Cells derived from 47 tumors of breast, ovarian, esophageal, and colorectal origin and 16 paired esophageal biopsies were exposed to anticancer agents (cisplatin; 5-fluorouracil; epirubicin; doxorubicin; paclitaxel; irinotecan and topotecan) in short-term cell culture (6 days). Real-time quantitative PCR was used to measure up- or down-regulation of 16 different resistance/target genes, and when tissue was available, immunohistochemistry was used to assess the protein levels.

**Results:**

In 8/16 paired esophageal biopsies, there was an increase in the expression of multi-drug resistance gene 1 (MDR1) following epirubicin + cisplatin + 5-fluorouracil (ECF) chemotherapy and this was accompanied by increased expression of the MDR-1 encoded protein, P-gp. Following exposure to doxorubicin *in vitro*, 13/14 breast carcinomas and 9/12 ovarian carcinomas showed >2-fold down-regulation of topoisomerase IIα (TOPOIIα). Exposure to topotecan *in vitro*, resulted in >4-fold down-regulation of TOPOIIα in 6/7 colorectal tumors and 8/10 ovarian tumors.

**Conclusion:**

This study suggests that up-regulation of resistance genes or down-regulation in target genes may occur rapidly in human solid tumors, within days of the start of treatment, and that similar changes are present in pre- and post-chemotherapy biopsy material. The molecular processes used by each tumor appear to be linked to the drug used, but there is also heterogeneity between individual tumors, even those with the same histological type, in the pattern and magnitude of response to the same drugs. Adaptation to chemotherapy may explain why prediction of resistance mechanisms is difficult on the basis of tumor type alone or individual markers, and suggests that more complex predictive methods are required to improve the response rates to chemotherapy.

## Background

Tumor resistance to chemotherapy is a well-known clinical phenomenon that is now yielding its secrets to investigation at the molecular level in biopsy material. Studies in cell lines do not always correlate well with results from tumor tissue [1], which may consist largely of non-neoplastic cells that support and modify the biology of neoplastic cells. Thus it is important to validate the mechanisms important *in vitro *with the situation in the patient. Nevertheless, cell line studies and immunohistochemical studies of tumors suggest that resistance is a selective process: only those cells that survive a drug-induced insult will re-grow.

We have previously shown development of such resistance to combination chemotherapy in tumor-derived cells from matched biopsies collected from breast cancer patients before and after administration of doxorubicin-containing chemotherapy [2]. In this study we show similar results in patients with esophageal cancer from biopsies obtained prior to and several months after chemotherapy. Two cycles of the combination of epirubicin, cisplatin and 5-FU (ECF) are given to these patients prior to resection, allowing studies to be performed with paired samples before and after chemotherapy. We have used real-time quantitative RT-PCR (qRT-PCR) and immunohistochemistry (IHC) to assess targets known to be of importance to resistance to these agents.

The mechanisms involved in resistance to chemotherapy usually involve up-regulation of resistance mechanisms, or down-regulation of target genes. Examples of the former include drug efflux pump molecules such as multi-drug resistance gene 1/P-glycoprotein (MDR1/P-gp), while the latter include topoisomerases (TOPOs), targets of drugs such as etoposide and doxorubicin. Many papers attest to the importance of clonal selection in this process: it is for instance possible to expose cell lines to low concentrations of drugs and, over time, to produce highly resistant sub-clones [3]. However, there is another potential mechanism that does not require clonal selection: cells may be able to adapt by regulation of expression of resistance or target molecules individually if they survive the initial exposure to the drug. This could be a more rapid process and would require changes in molecular expression, possibly due to epigenetic change, rather than genetic mechanisms such as mutation [4]. As a result, resistance may therefore arise rapidly following treatment with chemotherapy.

Recent studies have shown that the expression of MDR1/P-gp is up-regulated within hours of anti-cancer drug treatment *in vivo *in patient samples [5-8], although this effect was not observed in all patients. We therefore wished to examine how quickly and in how many cases these resistance molecules were up-regulated in tumor-derived cells from several tumor types. We have used selective short-term cell culture (6 days) to examine the changes in expression that occur following exposure to chemotherapy compared to medium-only control cells from the same samples. Our short-term culture system employs a serum-free medium and polypropylene 96 'U' well microplates. This inhibits the proliferation and survival of normal cells and allows selective survival of a neoplastic cell population [9]. The short incubation period also limits the possibility of selection of clones or sub-populations *in vitro*. However, the presence of non-neoplastic cells for most of the incubation period allows the interaction between stromal cells and neoplastic cells, a factor that appears to be important to maintain the chemosensitivity profile [10].

## Methods

### Patients and tissue samples for *in vitro *studies

Tumor derived cells were obtained from 17 breast cancer patients (16 primaries; 1 pre-treated with mitoxantrone and paclitaxel), 13 ovarian cancer patients (all pre-treated with a cisplatin-based regimen), 10 colorectal cancer patients (all primaries) and 7 esophageal cancer patients (3 untreated; 4 treated with ECF). Cells were grown for 6 days in serum-free medium with or without drugs, before RNA extraction and further PCR analysis.

### Patients and tissue samples for *in vivo *study

Thirty-four esophageal adenocarcinoma biopsies, thirty-two of which were paired samples were obtained from patients (17M:1F; median age 57, range 42–81) before and after administration of 2 cycles of ECF chemotherapy. After enzymatic digestion, tumor derived cells were centrifuged over Ficoll (Sigma Chemical Co, Poole, UK, Cat. No. 1077-1) to remove blood contaminating cells, washed in PBS, and stored in RNA later (Ambion, Huntingdon, UK) at -80°C until further molecular analysis was performed. Tissue sections from these samples were stained for GST-π, MRP1, P-gp and TS. All tumor samples were removed as part of patient treatment, with consent for tissue donation and local research ethics committee approval for use of the tissue surplus to diagnostic requirements for cellular and molecular assays. Chemosensitivity data were available for 9 patients with recurrent ovarian cancer, before treatment and on relapse, though in only one case was material available from both samples for quantitative RT-PCR.

### Drugs

Cisplatin, 5-fluorouracil (5-FU), epirubicin, doxorubicin, irinotecan, paclitaxel (Taxol^®^) and topotecan (Hycamptin^®^) were obtained from the pharmacy at Queen Alexandra Hospital (Portsmouth, UK). Cisplatin, 5-FU, irinotecan and paclitaxel were stored at room temperature, while all other drugs were stored at -20°C, as previously reported [11]. Test drug concentrations (TDC) were 10.0 μM for cisplatin, 345 μM for 5-FU, 148 μM for irinotecan, 15.9 μM for paclitaxel, 2.5 μM for doxorubicin, 0.862 μM for epirubicin, and 1.64 μM for topotecan. Combinations were made up by adding two or three drugs concurrently at their 200% TDC at the beginning of the ATP-cell viability assay and diluted in a constant ratio: sequential studies were not performed.

### Short-term cell culture

Briefly, tumor tissue or fluid was taken by a histopathologist or surgeon under sterile conditions, and transported to the laboratory in cell culture medium of Dulbecco's modified Eagle's medium (DMEM; Sigma Cat No. D5671) with antibiotics (100 U/ml penicillin and 100 μg/ml streptomycin, Sigma, Cat No. P0781) at 4°C. Cells were obtained from solid tumors by enzymatic dissociation, usually 0.75 mg/ml collagenase (Sigma Cat No. C-8051) overnight. Viable tumor-derived cells were purified by density centrifugation (Histopaque 1077-1, Sigma), washed, counted and resuspended to 100,000 cells/ml in case of effusions or 200,000 cells/ml for solid biopsies. In the meantime 96-well polypropylene microplates (Corning-Costar, High Wycombe, UK; Cat No. 3790) were prepared with each drug/combination at six doubling dilutions in triplicate from 200% TDC to 6.25% TDC, according to Andreotti *et al*. [9]. Approximately 10,000–20,000 cells/well were added to the plates to a final volume of 200 μl/well. The plates were then incubated at 37°C in 5% CO_2 _for 6 days, after which the degree of cell inhibition was assessed by measurement of the remaining ATP in comparison with negative control (no drug, MO) and positive control (maximum inhibitor, MI) rows of 12 wells each. Prior to cell lysis with an ATP-extracting reagent, an aliquot of 150 μl of cell suspension were removed from each well, centrifuged, washed with phosphate buffered saline (PBS) and stored at -80°C in a GTIC-containing solution (lysis buffer RA1, Macherey-Nagel, Düren, Germany; Cat. No.740961) until further molecular analysis was carried out. The RNA was subsequently extracted from aliquots that had been exposed to a drug concentration capable of inhibiting cell growth by 40–60%. ATP was extracted from the remaining 50 μl cell suspension and measured by light output in a microplate luminometer (Berthold Diagnostic Systems GmbH, Pforzheim, Germany) following addition of luciferin-luciferase.

### RNA extraction

Cells obtained after enzymatic dissociation from endoscopic esophageal biopsies or short term cell culture were either resuspended in RNA later (Ambion, Huntingdon, UK; Cat No. 7020) or lysed with buffer RA1 and stored at -80°C until RNA extraction. Total RNA was extracted from at least 50,000 cells with a commercially available kit (NucleoSpin^® ^RNA II mini, Macherey-Nagel; Cat No. 740955) according to the manufacturer's instructions. The protocol included a DNase digestion step to prevent carry-over of genomic DNA in further analysis.

### qRT-PCR

A two-step protocol was employed. Firstly, total RNA was reverse-transcribed using the Promega reverse transcription system (Promega, Southampton, UK; Cat No. A3500) including 8 μl RNA, 0.5 μg random primers, 20 units of recombinant RNasin^® ^ribonuclease inhibitor and 15 units of reverse transcriptase AMV (Promega, Cat No. M9004) to each 20 μl reaction. The resulting c-DNA was amplified by qPCR on a Biorad iCycler instrument (BioRad Laboratories, Hemel Hampstead, UK). The constituents of each PCR reaction (25 μl) were 1 μl of cDNA (or H_2_O), 200–500 nM of each primer (Table 1), 200 μM each dATP, dCTP, dGTP, 400 μM dUTP, 3.0–5.0 mM MgCl_2_, 0.125 units AMPErase^® ^UNG, 0.625 units of AmpliTaq Gold DNA polymerase and 1 × SYBR Green PCR buffer (all reagents were from Applied Biosystems, Warrington, UK). Product amplification was performed up to 45 PCR cycles, after uracil removal (2 min at 50°C) and polymerase activation (10 min at 95°C). Each two-step PCR cycle comprised denaturing (15 s at 95°C), annealing, and extending (1 min at 60°C). At the end of each run a final melt curve cycle (cooling to 50°C and then increasing stepwise 1°C to 95°C) was performed to exclude the presence of primer-dimer artefacts. At least 3 housekeeping genes were used for each experiment chosen from the following: glyceraldehyde-3 phosphate dehydrogenase (GAPDH), hypoxanthine phosphoribosyltransferase 1 (HPRT1), human porphobilinogen deaminase (PBGD), succinate dehydrogenase complex-subunit A (SDHA) and TATA box binding protein (TBP). The internal reference genes were selected due to their relative low abundance in normal tissue [12]. The housekeeping genes were amplified parallel to the target genes in separate wells. When possible, primer sequences (Table 1) were chosen to span exon boundaries to the following targets: breast cancer resistance protein (BCRP), dihydropyrimidine dehydrogenase (DPD), epidermal growth factor receptor (EGFR), excision repair cross-complementing 1 (ERCC1) gene, glutathione-S-transferase isoform π (GST-π), multi-drug resistance gene 1 (MDR1), MLH1, multi-drug resistance related protein 1 (MRP1), multi-drug resistance related protein 2 (MRP2), metallothionein (MT), major vault protein (MVP), thymidine phosphorylase (TP), thymidylate synthase (TS), topoisomerase I (TOPO I), topoisomerase IIα and β (TOPO IIα and TOPO IIβ). Each amplicon was amplified in a separate reaction to prevent competition among multiple sets of primers. A positive control (pooled c-DNA from a variety of human tumors, including breast, ovarian, colorectal and esophageal carcinoma) and negative controls with no template and RT-negative as template were added in every experiment. All assays were run in triplicate. Validation experiments were run to show that the efficiencies of the target and reference genes amplifications were approximately equal, and in the range 95–105%. The PCR cycle number that generated the first fluorescence signal above a threshold (threshold cycle, Ct; 10 standard deviations above the mean fluorescence generated during the baseline cycles) was determined, and a comparative Ct method was then used to measure relative gene expression [13]. The following formula was used to calculate the relative amount of the transcript in the sample: 2^-ΔΔCt^, where ΔCt is the difference in Ct between the gene of interest and the mean of the at least two reference genes, and ΔΔCt = ΔCt of drug non-exposed cells – ΔCt of drug-exposed cells, for the *ex-vivo *experiments, or ΔΔCt = ΔCt of pre-chemotherapy sample – ΔCt of post-chemotherapy sample, for matched tumor biopsies.

### Immunohistochemistry

The monoclonal antibodies (Abs) for GST-π, MRP1, Pg-p, and TP (Table 2) were detected using the Chemicon IHC Select™ – Immuno Peroxidase secondary detection system (Chemicon International, Chandlers Ford, Southampton, UK, Cat# Det-HP1000) and stained with 3,3' diaminobenzidine (DAB; HD Supplies, Aylesbury, UK, Cat. 4170) on a Dako Autostainer instrument (Dako, Ely, UK). Positive and negative controls were included with each run. Volumes of 50 μl avidin/ml goat serum and 50 μl biotin/ml primary Ab were used to block endogenous avidin binding. The slides were counterstained with Gills Haematoxylin, dehydrated and cleared using the Leica^© ^XL slide staining machine and mounted in Styrolite^® ^mounting medium (BDH, Poole, Dorset, UK; Cat No. 361704Y).

### Data analysis

The luminometer readings obtained from the ATP-TCA were entered into an Excel 2000 spreadsheet (Microsoft) which calculated the percentage of cell inhibition for each drug concentration according to the previous published formula: 1 - [(Test - MI)/(MO - MI)]*100 [9]. For each drug-response curve, the 50% inhibitory concentration (IC50) and the 90% inhibitory concentration (IC90) were also calculated as previously described [14].

Assessment of slides was done using the H-score. Staining intensity (none, 0 points; weak, 1 point; moderate, 2 points; strong, 3 points) and percentage of positive tumor cells were multiplied to achieve a score between 0 and 300. A H-score of 100 or more was regarded as positive and results less than 100 were regarded as negative. The correlation coefficients were calculated by the method of the least squares, and the correlation between the IC90 and IC50 values and immunohistochemistry indices was assessed using univariate linear regression (Statsdirect, Sale, UK).

Non-parametric statistical methods were used. The calculated and descriptive data were entered into an Access 2000 database (Microsoft) and analysed using a Wilcoxon two-tailed paired rank sum test for paired data or the Mann-Whitney U test for unpaired data, as appropriate (Statsdirect). IC50 and IC90 values for each compound were correlated to the relative mRNA levels of target genes using Spearman's rank correlation coefficient and multivariate analysis. On statistical advice, we chose not to use a Bonferroni's correction, but it should be noted that some technically statistically significant results could have arisen by chance.

## Results

### Chemotherapy-induced changes in mRNA levels in biopsy material

**Figure 1:**
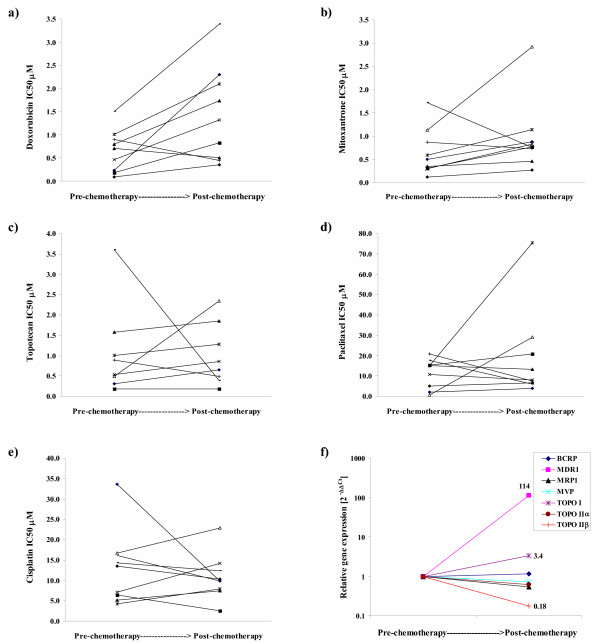
Changes due to chemotherapy in biopsies taken before and after chemotherapy. Cytotoxicity of a) doxorubicin; b) mitoxantrone; c) topotecan; d) paclitaxel and e) cisplatin (expressed as IC50 μM) in paired samples obtained from ovarian cancer patients before and after they were treated with an anthracycline containing regimen (paclitaxel+mitoxantrone n = 7; liposomal doxorubicin n = 2). Each line represents an individual patient. (f) Gene expression changes pre- and post-treatment (paclitaxel plus mitoxantrone chemotherapy) in one patient were analysed by qRT-PCR following *in vitro *exposure to doxorubicin.

Figures 1a–e illustrate changes to IC50 μM values in pre- and post chemotherapy ovarian tumor-derived cells tested with a variety of chemotherapeutic agents. There is a general increase in resistance to doxorubicin when anthracycline-based regimens (mitoxantrone + paclitaxel or liposomal doxorubicin) are administered to ovarian cancer patients (Fig. 1a). We observed little cross-resistance for mitoxantrone, topotecan, paclitaxel, and cisplatin following anthracycline-based chemotherapy (Fig. 1b–e), [15], although some patients do show alteration of sensitivity to other agents. Similar changes can be seen in breast cancer biopsies before and after administration of a chemotherapy regimen [2]. The increase in resistance to doxorubicin shown in Figure 1a was accompanied by changes in drug resistance gene expression. Figure 1f highlights such up-regulation of MDR1 and breast cancer resistance protein (BCRP) observed in ovarian-tumor derived cells from a single patient.

**Figure 2:**
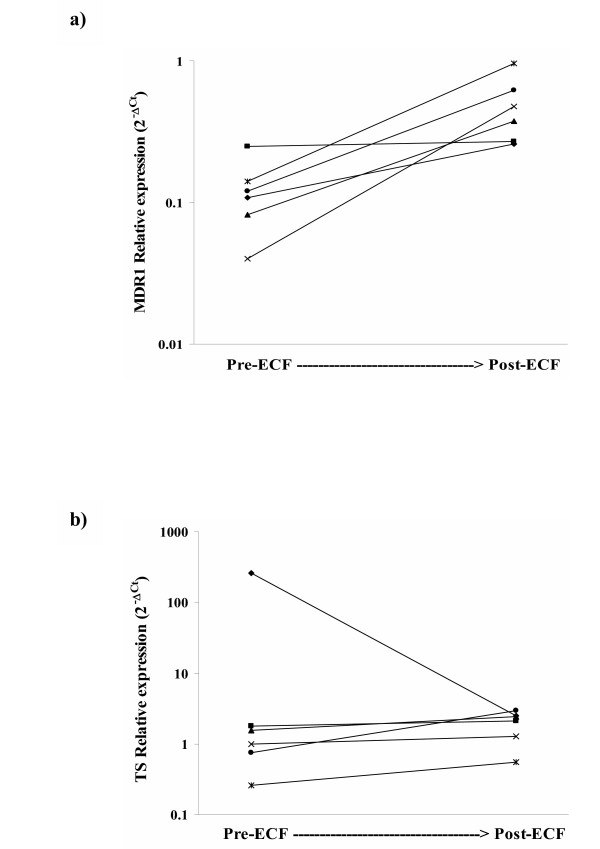
Changes in relative gene expression in 6 paired esophageal tumor biopsies. a) Changes in MDR1 relative gene expression and b) changes in TS relative gene expression. Each dot represents the relative mRNA level for an individual tumor, measured before and after ECF chemotherapy.

Membrane proteins such as MDR1/P-gp, multi-drug resistance protein 1 (MRP1) and major vault protein (MVP) have been demonstrated to confer resistance to epirubicin by pumping the cytotoxic drug out of cells [16]. Paired esophageal samples were obtained from the same six patients both before and after chemotherapy; in all 6 biopsy pairs (Table 3), there was an increase of the mRNA levels of MDR1 following ECF chemotherapy (Fig. 2a). In a larger series of 20 esophageal samples (including those that did not have a paired biopsy), divided in two groups according to the patients' exposure to treatment, there was a 5-fold increase in MDR1 expression in the post-chemotherapy group (median expression index 0.07 pre- and 0.37 post-chemotherapy, p < 0.0001, Mann-Whitney non-parametric unpaired U test). The paired samples from patients before and after ECF chemotherapy also showed a trend towards increased expression of MRP1 and MVP (Table 3), with three tumors showing concomitant up-regulation of both genes. Furthermore, the increased expression of MRP1 paralleled that of glutathione-S-transferase isoform π (GST-π) in 2 of these 3 samples, consistent with the mechanism of detoxification of MRP1 that involves transport of glutathione-conjugated molecules [17].

We then determined the relative mRNA levels of enzymes that have previously been correlated with 5-FU sensitivity: dihydropyrimidine dehydrogenase (DPD), thymidine phosphorylase (TP) and thymidylate synthase (TS) [18]. No significant difference was found for these 3 genes when we compared their expression pre- and post-chemotherapy in unpaired samples (Table 3). However, we found a modest increase of TS expression in 5/6 paired samples (Fig. 2b), 3 of which also showed a concomitant decrease in TP levels. One sample showed a paradoxical decrease in TS, indicating some heterogeneity. Although the sample size is small, the trend of increased TS levels in ECF exposed tumors is consistent with a number of previous reports that demonstrated an acute induction of TS expression in cell lines, animal models and human tumors following 5-FU treatment [18,19].

It should be noted that these qRT-PCR results were all obtained from samples that included normal cells present in the tumor as well as neoplastic cells, and could be affected by changes in normal cells as well as malignant cells.

### Chemotherapy-induced changes in protein levels in biopsy material

IHC was performed on 16 paired esophageal biopsies: qRT-PCR data were obtained for four of these pairs. Overall, we were able to confirm an increased expression of the MDR-1 encoded protein, P-gp (Table 4), in 8/16 paired tumor samples obtained from patients who had been exposed to ECF chemotherapy. TS positivity (Table 4) was found in 10/16 samples obtained before treatment and 10/16 post-chemotherapy specimens; in this instance the modest induction of TS seen at the mRNA level did not reflect an increase in protein level (data not shown). We found GST-π (Table 4) positivity in 5/16 pre-chemotherapy biopsies. In the post-chemotherapy specimens we detected strong expression of GST-π in all but one of the samples. In one case that had been negative at diagnosis we measured a marginal increase of mRNA levels with qRT-PCR. A total of 13/16 pre- and 12/16 post-chemotherapy biopsies were found to be positive for MRP1 (Table 4). Overall, these data show little correlation between the mRNA and the degree of expression of protein levels determined by IHC. Confounding factors may be the presence of non-cancerous cells as mentioned above, but also the nature of IHC, which is at best a semi-quantitative technique.

### Changes in short-term cell culture

Of the 47 solid tumor samples studied in short-term cultures, 7 were esophago-gastric, 17 were breast carcinomas, 13 were ovarian carcinomas and 10 were colorectal tumors. A total of 93 experiments were performed, as in most cases the same sample was treated with 2 or more drugs.

### Doxorubicin effects in short-term cell culture

**Figure 3:**
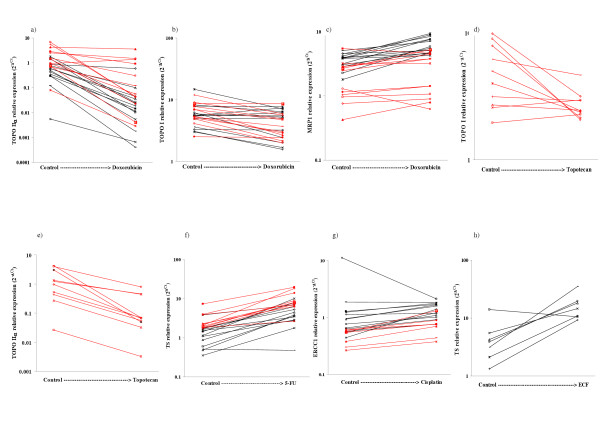
Changes in relative expression of putative chemoresistance genes in tumor-derived cells. Each dot represents the relative mRNA level for an individual sample, measured after *in vitro *drug exposure compared with untreated control cells. (a) TOPO IIα expression in breast (black lines) and ovarian (red lines) tumor cells after doxorubicin exposure. (b) TOPO I expression in breast (black lines) and ovarian (red lines) tumor cells after doxorubicin exposure. (c) MRP1 expression in breast (black lines) and ovarian (red lines) tumor cells after doxorubicin exposure. (d) TOPO I expression in ovarian tumor cells after topotecan exposure. (e) TOPO IIα expression in ovarian tumor cells after topotecan exposure. (f) TS expression in breast (red lines) and colorectal (black lines) tumor cells after 5-FU exposure. (g) ERCC1 expression in breast (black lines) and ovarian (red lines) tumor cells after cisplatin exposure. (h) TS expression in esophageal tumor cells after ECF exposure. The numerical data for these graphs is summarized in Tables 5-8.

In the first instance, we decided to study the effects of anthracycline exposure for 6 days on tumour-derived cells. Our *in vitro *experiments showed a significant down-regulation of TOPO IIα; its median levels decreased from 0.546 (range 0.005–1.782) to 0.017 (range 0.0004–0.595) units in a group of 14 breast samples (p < 0.0001, Wilcoxon matched pairs test) and from 1.491 (range 0.080–6.884) to 0.089 (range 0.003–3.605) units in the ovarian cancer subgroup (p < 0.0015, Wilcoxon matched pairs test). The down-regulation of TOPO IIα levels was greater than 2-fold in 13/14 breast samples and in 9/12 ovarian samples (Fig. 3a). It has been suggested that cancer cells can concomitantly down-regulate TOPO IIα and up-regulate TOPO I in response to treatment with topoisomerase II inhibitors [20]. In our series we noted a general trend of diminished levels of TOPO I, particularly in the ovarian subgroup (Fig 3b). It is possible that doxorubicin exposure resulted in quiescence of cells which were not killed, and which would not therefore require higher levels of TOPO I, or that doxorubicin may also partly inhibit TOPO I, as suggested by a previous study [21].

We observed a significant up-regulation of MRP-1 (p < 0.0001, Wilcoxon matched pairs test; Fig. 3c) and of MVP (p < 0.0023, Wilcoxon; Table 5) in the breast cancer subgroup. The same effect was not found in the ovarian cancer group, in which the levels of MRP-1 and MVP increased over 0.5 fold in only 4/12 and 3/12 samples, respectively (Table 5). No significant changes were found in the expression of MDR-1 and BCRP, although it should be noted that there was substantial heterogeneity among individual tumors (Table 5). For example, we measured a greater than 2-fold increase of MDR-1 levels in 2/12 ovarian samples (none of whom had received a MDR-1 pumped chemotherapeutic agent before), and a significant up-regulation of BCRP in 3/12 ovarian samples.

### Topoisomerase I inhibitors effects in short-term cell culture

Subsequently we looked at the effect of 2-camptothecin derivatives, irinotecan and topotecan, on colorectal and ovarian tumor cells, respectively (Table 6). As both compounds act by TOPO I inhibition, we firstly measured the levels of their target enzyme. We noted a trend towards down-regulation of TOPO I in treated cells: exposure to irinotecan decreased TOPO I levels >2-fold in 3/7 colorectal tumors, while topotecan caused down-regulation in 4/10 ovarian samples (Fig. 3d). The decrease of TOPO I was accompanied by a concomitant reduction of TOPO IIα expression, which was particularly pronounced (>4-fold) in 6/7 colorectal tumors and 8/10 ovarian tumors (Fig. 3e).

No significant changes were observed in the expression of the drug efflux molecules, MDR-1, BCRP and MRP-1 (Table 6), though, as in the case of doxorubicin, considerable heterogeneity was noted. We observed an increase of BCRP levels after irinotecan or topotecan exposure in 2/7 colorectal samples and 3/9 ovarian samples.

Amongst the genes implicated in DNA repair (Table 6), the modest down-regulation of MLH1 by topotecan exposure was not found statistically significant, although it was noted in 7/10 ovarian samples. Up-regulation of ERCC1 expression was found in all 10 ovarian cancer samples exposed to topotecan (p < 0.002, Wilcoxon), and in all 7 colorectal specimens treated with irinotecan, although in this group the increase was modest (p = 0.016, Wilcoxon).

Finally, we looked at EGFR as a marker of tumor growth and progression. After topotecan exposure we found a decrease (more than 2-fold) in the expression of this growth factor in 9/10 ovarian samples (Table 6), with the median levels decreasing from 0.470 to 0.163 units (p < 0.0039, Wilcoxon).

### 5-FU effects in short-term cell culture

Preliminary data obtained from 2 esophageal biopsies indicated an increase of TS levels following 5-FU treatment. Subsequent *in vitro *experiments performed on material derived from 10 colorectal tumor samples and 13 breast tumor samples confirmed the expected increase of TS levels (Table 7) in the samples exposed to 5-FU (p < 0.0001, Wilcoxon; Fig. 3f). The results indicated a general trend towards DPD down-regulation in the cells that had been exposed to 5-FU *in vitro*, though this effect was modest in most cases and we observed a greater than 2-fold decrease in only 7/13 breast samples and 2/10 colorectal samples.

### Cisplatin effects in short-term cell culture

We examined the mRNA expression levels of genes previously highlighted as important in cisplatin resistance in tumor-derived cells from 13 breast and 7 ovarian tumors (Table 8). Up-regulation of the nucleoside excision repair (NER) gene ERCC1 has previously been shown to increase the removal of platinum affected DNA [22]. We observed a significant increase in ERCC1 levels in both tumor-derived breast (p = 0.0266, Wilcoxon matched pairs test) and ovarian cells (p = 0.0156, Wilcoxon matched pairs test) (Fig 3g). We also observed significant down regulation of a second DNA repair gene, MSH2 in tumor-derived breast cells (p = 0.0479, Wilcoxon matched pairs test), but not in tumor-derived ovarian cells. Samimi *et al*., [23] have previously demonstrated that following cisplatin therapy, there is selection for cells expressing lower hMLH1 and hMSH2. However, no significant changes were observed in the other mismatch repair genes, MLH1 and MSH6 in either tumor type (Table 8).

There was no significant change in MGMT in tumor-derived breast or ovarian cells. However, it has previously been shown that MGMT mRNA levels begin to recover after 24 hours in the absence of drug [24]. There was a paradoxical decrease in the copper export pump ATP7B following cisplatin exposure (Table 8), in tumor-derived breast cells (p = 0.0327, Wilcoxon matched pairs test), but not in ovarian cells. No significant changes were observed in the heavy-metal binding protein MTII, in either tumor type.

### ECF effects in short-term cell culture

We were able to study tumor-derived cells from 7 esophageal cancer patients, 4 of which had already been given ECF *in vivo*. The results mirror those obtained from paired esophageal biopsies: we noticed increased expression of TS in the cells that had been exposed to ECF (Fig 3h). However, the expected up-regulation of MDR1 was only detected in 4/7 samples (data not shown), and may therefore be occurring in non-neoplastic cells that do not normally express MDR1, rather than in the neoplastic cells, which we found to express this molecule to the same degree pre- and post-treatment.

### Correlation of *in vitro *cytotoxicity with molecular expression

Lastly, for 5-FU, doxorubicin and irinotecan, IC90 and IC50 data were obtained by measuring the ATP levels in a small aliquot of cell suspension at the end of the incubation period. This allowed a comparison to be made between drug sensitivity and the expression of putative resistance genes in the cells that had been exposed to chemotherapeutic agents. Using multivariate analysis, the IC90 of 5-FU was correlated with the median change of mRNA levels of both TS and DPD measured in 5-FU treated tumor-derived cells compared to control cells (expressed as 2^-ΔΔCt^) (R^2 ^= 0.872704; p = 0.0006 for DPD; p < 0.0001 for TS). In addition, expression of ERCC1 mRNA correlated with the IC50 values determined for doxorubicin in 11 breast samples (R = 0.7204, p < 0.0124). No other correlations were noted between sensitivity to the drugs and gene expression levels.

## Discussion

Our results suggest that rapid adaptation to chemotherapy may result in a resistant phenotype. This is mediated by down- or up-regulation of genes that are usually correlated to the mechanism of action of the individual chemotherapeutic agent or relevant resistance mechanisms. Our data suggest that short-term cell culture of tumor-derived cells with drugs could provide a suitable model for studying resistance mechanisms. The mechanisms observed appeared to be more specific to the drug used than to the tumor type: constitutive resistance probably reflects pre-chemotherapy expression of resistance mechanisms (e.g. drug efflux molecule expression in esophageal carcinoma). Acquired resistance develops rapidly and is likely to reflect changes in gene regulation rather than mutation-dependent selection of clones. Clonal selection may be important in some rapidly growing tumors and cell lines grown in serum-containing media, but is unlikely to be the major factor in solid tumors which have relatively low doubling rates. While mutation-mediated resistance can be much more profound than that observed here, our data suggest that the functional effects may still be sufficient to render the patient's tumor resistant to treatment within one cycle of chemotherapy.

### Changes in resistance and target molecules

A large proportion of the published studies on resistance to chemotherapy have investigated the development of resistance using cell lines generated in the lab after prolonged and step-wise exposure to anti-cancer drugs. These *in vitro *models are not necessarily representative of the *in vivo *situation, when patients are usually administered one cycle of chemotherapy every 3–4 weeks. There are few studies in clinical samples. Our approach allows us to expose the tumor cells to single drugs under carefully controlled conditions, even if this would be an inappropriate drug for that particular patient. We are then able to look concomitantly at cytotoxicity, and molecular markers of resistance in the same experiment.

Anthracyclines have a mechanism of action that includes TOPO IIα inhibition *via *DNA intercalation. Resistance to anthracyclines is thought to be mediated by a number of different mechanisms, which include mutation or alteration of its target enzyme, TOPO IIα, and up-regulation of drug efflux proteins, such as BCRP, MRP1, MVP and MDR-1 [25]. Our data show that many of the cell line data are correct: in most solid tumors, it appears that anthracycline and topotecan exposure do lead to decreased topoisomerase II and I expression respectively, while inducing the expression of drug efflux pump molecules. The reason for the alteration in topoisomerase II expression following topotecan exposure is not clear, but topotecan does affect cell proliferation, and any reduction in proliferation would indirectly affect the expression of topoisomerase II alpha induced during S phase. However, our results also suggest that the heterogeneity of chemosensitivity between tumors is reflected by heterogeneity of molecular determinants of resistance/sensitivity.

The increased TS levels in ECF and 5-FU exposed cells is consistent with a number of previous reports. Gene amplification of TS with consequent increases in TS mRNA and protein has been observed in cell lines that are resistant to 5-FU and fluorodeoxyuridine (FUDR) [26,27]. Treatment with 5-FU has been shown to acutely induce TS expression in cell lines, animal models and human tumors [19,28-30]. In general there is strong evidence that the expression of DPD and TS in GI cancers is predictive of response to 5-FU. It should be noted that the concomitant measurement of both these markers markedly enhanced the ability to predict tumor response to 5-FU-based chemotherapy in a number of studies [31-34].

### Gene profiling and drug response

There are few studies comparing gene expression before and after chemotherapy. A few studies have reported microarray data in biopsy material taken before and after (or even during) chemotherapy. These studies are of great interest, though normal cell effects cannot be excluded from the results. Buchholz *et al*. [35] employed cDNA microarray to measure gene expression changes during chemotherapy in 5 patients with breast cancer. Clarke *et al*. [36] studied gene expression changes in 18 rectal cancer patients undergoing therapy with Mitomycin C or 5-FU. This study reported a number of genes implicated in protein synthesis and RNA metabolism to be significantly decreased during drug treatment. These studies are not directly comparable: tumors of different types respond better to different drugs, and differences in their adaptation to these drugs are therefore expected on the basis of their innate sensitivity or resistance.

### The potential for positive selection: molecular chess

It is common to show a cross-over effect with clinical trials of treatments with differing mechanisms of action, in which patients treated with one type of chemotherapy show sensitivity to the alternative regimen following failure of the one to which they were allocated. The recognition that selection of a molecular phenotype by exposure to one anti-cancer agent may leads to the expression of molecular targets for other drugs raises the possibility that it might be possible to enhance sensitivity to second-line or maintenance therapy by careful selection of patients for first-line therapy [37]. This approach would provide patients with a "backstop" for their first-line chemotherapy. One can envisage a series of interlocking treatments using drugs with specific molecular targets, monitored by molecular assays, which would allow the oncologist to employ a form of molecular chess to defeat the tumor. This approach might overcome the inherent heterogeneity, which is likely to underlie the variable results obtained from sequential chemotherapy to date. Assessment of this process in tumors could provide predictive assays allowing the oncologist to tailor therapy to the patient and avoid the development of resistance within the tumor.

## Conclusion

In summary, this study suggests that up-regulation of resistance genes or down-regulation in target genes may occur rapidly in human solid tumors, within days of the start of treatment, and that similar changes are present in pre- and post-chemotherapy biopsy material. The molecular processes used by each tumor appear to be linked to the drug used, but there is heterogeneity between individual tumors, even those with the same histological type, in the pattern and magnitude of response to the same drugs. Adaptation to chemotherapy may explain why prediction of resistance mechanisms is difficult on the basis of tumor type alone or individual markers, and suggests that more complex predictive methods are required to improve the response rates to chemotherapy.

## List of abbreviations

Gene names were abbreviated as follows: glyceraldehyde-3 phosphate dehydrogenase (GAPDH), hypoxanthine phosphoribosyltransferase 1 (HPRT1), human porphobilinogen deaminase (PBGD), succinate dehydrogenase complex-subunit A (SDHA) and TATA box binding protein (TBP), breast cancer resistance protein (BCRP), dihydropyrimidine dehydrogenase (DPD), excision repair cross-complementing 1 (ERCC1) gene, epidermal growth factor receptor (EGFR), glutathione-S-transferase isoform π (GST-π), multi-drug resistance gene 1 (MDR1), mutL homologue 1 (MLH1), multi-drug resistance related protein 1 (MRP1), multi-drug resistance related protein 2 (MRP2), metallothionein II (MT II), major vault protein (MVP), thymidine phosphorylase (TP), thymidylate synthase (TS), topoisomerase I (TOPO I), topoisomerase IIα and β (TOPO II).

## Competing interests

IAC is a director of CanTech Ltd. The remaining authors declare that they have no competing interests.

## Authors' contributions

IAC, FDN and SJM conceived and designed the study. FDN, SJM, LAK, PAW, SS, AF and SG participated in the short-term cell culture studies. FDN, SJM, LAK and FGG also carried out the qRT-PCR studies. PJ carried out the immunohisotochemical studies and IAC and SDP carried out histological analysis. IAC, FDN, SJM and BH participated in the statistical analysis. All authors participated in the data analysis, drafting of the manuscript and read and approved the final version.

## Pre-publication history

The pre-publication history for this paper can be accessed here:



## Figures and Tables

**Table 1:** List of primers for qRT-PCR. Sequence of primers (forward and reverse) used for qRT-PCR experiments. GenBank accession numbers for each gene are indicated in brackets. The primers were designed using an old version of the software Primer 3.0, available at the following website:

**Name**	**Sequence 5'-3' (forward and reverse primer)**	**Product length**	**Primer nM**	**Mg^2+ ^mM**	**Reference**
GAPDH* (NM_002046)	GAA GGT GAA GGT CGG AGT C	226	200	4	-
	GAA GAT GGT GAT GGG ATT TC		200		
HPRT1* (NM_000194)	TCA GGC AGT ATA ATC CAA AGA TGG T	84	400	4	[38]
	AGT CTG GCT TAT ATC CAA CAC TTC G		400		
PBGD* (NM_000190)	CTG CAC GAT CCC GAG ACT CT	98	400	4	[39]
	GCT GTA TGC ACG GCT ACT GG		400		
SDHA* (NM_004168)	TGG GAA CAA GAG GGC ATC TG		400	4	[12]
	CCA CCA CTG CAT CAA ATT CAT G		400		
TBP* (X54993)	CAC GAA CCA CGG CAC TGA TT	89	400	4	[40]
	TTT TCT TGC TGC CAG TCT GGA C		400		
BCRP (AF098951)	CAC AAC CAT TGC ATC TTG GC	74	400	4	[41]
	GCT GCA AAG CCG TAA ATC CA		400		
DPD (NM_000110)	CCA AAG GCA GTA AAG CAG GAA	66	400	4	[42]
	TCA CGA CTC CCC GTA TCG A		400		
EGFR (NM_005228)	TGG TCA AGT GCT GGA TGA TAG A	156	400	4	-
	GGT AGA AGT TGG AGT CTG TAG GA		400		
ERCC-1 (NM_001983)	GGG AAT TTG GCG ACG TAA TTC	71	400	3	[43]
	GCG GAG GCT GAG GAA CAG		400		
GST-π (NM_000852)	CGG AGA CCT CAC CCT GTA	169	400	5	-
	CGC CTC ATA GTT GGT GTA GA		400		
MDR1 (AF016535)	TGG TTC AGG TGG CTC TGG AT	72	300	4	[44]
	CTG TAG ACA AAC GAT GAG CTA TCA CA		300		
MLH-1 (NM_000249)	GGC ACA GCA TCA AAC CAA GT	147	400	4	-
	GCA AGC ATG GCA AGG TCA A		400		
MRP1 (L05628)	CAA TGC TGT GAT GGC GAT G	70	400	4	[39]
	GAT CCG ATT GTC TTT GCT CTT CA		400		
MRP2 (NM_000392)	TGC AGC CTC CAT AAC CAT GAG	80	400	3	[45]
	GAT GCC TGC CAT TGG ACC TA		400		
MT-II (NM_005953)	GAT CCC AAC TGC TCC TGC	127	400	4	-
	ACT TGG CAC AGC CCA CAG		400		
MVP (NM_017458)	CAG CTG GCC ATC GAG ATC A	68	400	4	[39]
	TCC AGT CTC TGA GCC TCA TGC		400		
TP (NM_001953)	CCT TGG ATA AGC TGG AGT CT	107	400	4	-
	CCT ACT CTG ACC CAC GAT AC		400		
TS (NM_001071)	CCA GAG ATC GGG AGA CAT GG	66	400	4	-
	TAC GTG AGC AGG GCG TAG CT		400		
TOPO I (J03250)	CTC CAC AAC GAT TCC CAG AT	149	400	3	-
	TTA TGT TCA CTG TTG CTA TGC TT		400		
TOPO IIα (NM_001067)	GTA ATT TTG ATG TCC CTC CAC GA	223	400	3	-
	TCA AGG TCT GAC ACG ACA CTT		400		
TOPO IIβ (NM_001068)	GCA GCC GAA AGA CCT AAA TA	85	400	3	-
	AAT CAT TAT TGT CAT CAT CAT CAT C		400		

(N.B. Where primer sequences have been taken from references the PCR conditions have been further optimised compared to the original reference.)

* Housekeeping genes.

**Table 2:** List of antibodies used for immunohistochemical studies.

**Antibody**	**Pre-Treatment**	**Dilution**	**Incubation**	**Cat No.**	**Source**	**Control Tissue**
Glutathione S-Transferase pi GST-π (polyclonal)	None	1:150	30 min RT	PU249-UP	BioGenex (Distributor: Menarini Diagnostics, Wokingham, UK)	Breast Ca
P-glycoprotein (MDR-1) (Clone JSB-1)	Pressure Cook 2 min pH 6.0	1:100	Overnight 4°C	NCL-JSB1	Novo Castra Newcastle-upon-Tyne, UK	Kidney
Multidrug Resistant-Related Protein (MRP) (Clone MRPm6, specific for MRP-1)	Pressure Cook 2 min pH 7.0	1:30	30 min RT	MAB4122	Chemicon International Chandlers Ford, UK	Kidney
Thymidylate Synthase TS (Clone TS 106)	Pressure Cook 2 min pH 6.0	1:20	30 min RT	MS471P	Neo Markers (Distributor: Lab Vision, Newmarket, UK)	Colon Ca

When sections required microwaving a Matsui MIIOM microwave was used at 800 W power. Pressure cooking was performed with a Tefal Clipso Pressure Cooker using 70 P power.

**Table 3:** Relative expression of mRNA levels in esophageal samples obtained from patients before and after chemotherapy (median values). The last 2 columns on the right represent values for the 12 paired biopsies. The p values have been calculated using non parametric statistics, and in detail the Mann Whitney U test for unpaired samples, and the Wilcoxon matched pairs test for paired samples.

	**All esophageal samples**			**Paired esophageal samples**		
				
**Target gene**	**Pre-chemo n = 13**	**Post-chemo n = 6**	**p**	**Pre-chemo n = 6**	**Post-chemo n = 6**	**p**
DPD	0.71	1.09	0.4117	0.68	1.09	0.3750
GST-π	4.39	0.88	0.1969	1.61	1.85	0.8438
MDR1	0.07	0.37	**<0.0001**	0.11	0.43	**0.0313**
MRP1	4.81	10.60	0.1624	4.58	10.77	0.4375
MT II	36.35	23.43	0.5846	12.49	24.12	0.8438
MVP	6.61	8.57	0.6544	6.39	9.11	0.5625
TP	4.21	2.58	0.2326	3.56	3.01	>0.999
TS	1.11	2.43	0.1851	1.27	2.27	0.4375

**Table 4:** Median expression (range in brackets) of protein levels in paired esophageal samples (n = 16) obtained from patients before and after chemotherapy. Slides were assessed using the H-score. A H-score of 100 or more was regarded as positive and below 100 was regarded as negative.

**Antibody**	**Pre-chemotherapy**	**Post-chemotherapy**
GST-π	35 (0–300)	200 (70–300)
P-gP	45 (0–300)	100 (0–300)
MRP	100 (0–200)	100 (10–200)
TS	120 (0–300)	100 (0–300)

**Table 5:** Relative expression of mRNA levels in tumor samples after *ex vivo *exposure to doxorubicin. The IC_50 _concentrations for the samples tested are shown for each drug (median and range). The p values have been calculated using non-parametric statistics, in detail the Wilcoxon matched pairs test. On statistical advice, we chose not to use a Bonferroni's correction, but it should be noted that some technically statistically significant results could have arisen by chance.

	**Breast samples**				**Ovarian samples**			
	
**Target gene**	**n**	**Control**	**Doxorubicin **IC_50 _= 0.828 μM (0.569–1.16)	**p**	**n**	**Control**	**Doxorubicin **IC_50 _= 1.34 μM (0.310–17.7)	**p**
**BCRP**	14	0.039	0.044	0.8077	12	0.010	0.027	0.042
**ERCC1**	14	0.711	0.826	0.2412	11	0.440	0.506	0.4922
**MDR1**	14	0.069	0.056	0.0494	12	0.005	0.005	0.2036
**MRP1**	14	3.886	5.692	<0.0001	12	1.890	2.321	0.083
**MVP**	14	4.990	6.925	0.0023	12	2.251	2.687	0.042
**TOPO I**	14	5.464	5.128	0.0419	12	6.112	3.360	0.001
**TOPO IIα**	14	0.546	0.017	<0.0001	12	1.491	0.089	0.0015
**TOPO IIβ**	14	6.617	6.461	0.0785	12	5.907	5.252	0.0322

**Table 6:** Relative expression of mRNA levels in tumor samples after *ex vivo *exposure to topotecan and irinotecan. The IC_50 _concentrations for the samples tested are shown for each drug (median and range). The p values have been calculated using non-parametric statistics, in detail the Wilcoxon matched pairs test. On statistical advice, we chose not to use a Bonferroni's correction, but it should be noted that some technically statistically significant results could have arisen by chance.

	**Ovarian samples**				**Colorectal samples**			
	
**Target gene**	**n**	**Control**	**Topotecan **IC_50 _= 0.754 μM (0.164–4.82)	**p**	**n**	**Control**	**Irinotecan **IC_50 _= 55.4 μM (29.5–100)	**p**
**BCRP**	9	0.0156	0.0125	>0.999	7	0.0354	0.0515	0.6875
**COX-2**	10	0.253	0.475	0.7695	7	105.17	38.49	0.0156
**EGFR**	10	0.470	0.163	0.0039	-	-	-	-
**ERCC1**	10	0.252	0.399	0.002	7	1.403	2.469	0.0156
**MDR1**	9	0.0090	0.0034	0.5703	7	0.2253	0.2938	>0.999
**MLH1**	10	0.263	0.2268	0.0371	-	-	-	-
**MRP1**	-	-	-	-	7	5.132	3.775	0.0156
**TOPO I**	10	4.490	2.448	0.0371	7	1.468	1.119	0.1094
**TOPO IIα**	10	1.093	0.0670	0.002	6	0.2847	0.0733	0.2188
**TOPO IIβ**	-	-	-	-	7	0.7405	0.5058	0.0313

**Table 7:** Relative expression of mRNA levels in tumor samples after *ex vivo *exposure to 5 FU. The IC_50 _concentrations for the samples tested are shown for each drug (median and range). The p values have been calculated using non-parametric statistics, in detail the Wilcoxon matched pairs test. On statistical advice, we chose not to use a Bonferroni's correction, but it should be noted that some technically statistically significant results could have arisen by chance.

	**Breast samples **IC_50 _= 200 μM (86.5–363)				**Colorectal samples **IC_50 _= 112 μM (13.8–311)			
	
**Target gene**	**n**	**Control**	**5-FU**	**p**	**n**	**Control**	**5-FU**	**p**
**DPD**	13	1.289	0.494	0.0012	10	0.503	0.448	0.4922
**TP**	13	8.876	10.928	0.6848	10	4.616	5.963	0.084
**TS**	13	1.136	3.819	0.0005	10	2.157	7.954	0.002

**Table 8:** Relative expression of mRNA levels in tumor samples after *ex vivo *exposure to cisplatin. The IC_50 _concentrations for the samples tested are shown for each drug (median and range). The p values have been calculated using non-parametric statistics, in detail the Wilcoxon matched pairs test. On statistical advice, we chose not to use a Bonferroni's correction, but it should be noted that some technically statistically significant results could have arisen by chance.

	**Breast samples **IC_50 _= 18.4 μM (8.9–30.9)				**Ovarian samples **IC_50 _= 17.8 μM (6.3–25.6)			
	
**Target gene**	**n**	**Control**	**Cisplatin**	**p**	**n**	**Control**	**Cisplatin**	**p**
**ATP7B**	13	0.256	0.177	0.0327	7	0.268	0.111	0.4375
**ERCC1**	13	0.933	1.382	0.0266	7	0.542	0.758	0.0156
**MGMT**	13	1.203	1.275	0.8926	7	0.794	0.944	0.1094
**MLH1**	13	0.758	1.000	0.1465	7	0.512	0.683	0.3750
**MSH2**	13	1.289	1.047	0.0479	7	2.579	2.732	0.9375
**MSH6**	13	2.351	2.194	0.1909	7	2.997	2.194	0.1094
**MTII**	13	44.221	53.817	0.5417	7	12.996	11.445	0.8125
